# A wild ‘albino’ bilberry (*Vaccinium myrtillus* L.) from Slovenia shows three bottlenecks in the anthocyanin pathway and significant differences in the expression of several regulatory genes compared to the common blue berry type

**DOI:** 10.1371/journal.pone.0190246

**Published:** 2017-12-22

**Authors:** Zala Zorenc, Robert Veberic, Ana Slatnar, Darinka Koron, Silvija Miosic, Ming-Hui Chen, Christian Haselmair-Gosch, Heidi Halbwirth, Maja Mikulic-Petkovsek

**Affiliations:** 1 Department of Agronomy, Chair for Fruit, Wine and Vegetable Growing, Biotechnical Faculty, University of Ljubljana, Ljubljana, Slovenia; 2 Department of Fruit Growing, Viticulture and Oenology, Agricultural Institute of Slovenia, Ljubljana, Slovenia; 3 Institute of Chemical, Environmental and Biological Engineering, Technische Universität Wien, Vienna, Austria; Universidade de Lisboa Instituto Superior de Agronomia, PORTUGAL

## Abstract

Relative expressions of structural genes and a number of transcription factors of the anthocyanin pathway relevant in *Vaccinium* species, and related key enzyme activities were compared with the composition and content of metabolites in skins of ripe fruits of wild albino and blue bilberry (*Vaccinium myrtillus*) found in Slovenia. Compared to the common blue type, the albino variant had a 151-fold lower total anthocyanin and a 7-fold lower total phenolic content in their berry skin, which correlated with lower gene expression of *flavonoid 3-O-glycosyltransferase* (*FGT*; 33-fold), *flavanone 3-hydroxylase* (*FHT*; 18-fold), *anthocyanidin synthase* (*ANS*; 11-fold), *chalcone synthase* (*CHS*, 7.6-fold) and *MYBPA1* transcription factor (22-fold). The expression of *chalcone isomerase (CHI)*, *dihydroflavonol 4-reductase (DFR)*, *leucoanthocyanidin reductase* (*LAR*), *anthocyanidin reductase* (*ANR*) and *MYBC2* transcription factor was reduced only by a factor of 1.5–2 in the albino berry skins, while *MYBR3* and *flavonoid 3’*,*5’-hydroxylase* (*F3’5’H*) were increased to a similar extent. Expression of the SQUAMOSA class transcription factor *TDR4*, in contrast, was independent of the color type and does therefore not seem to be correlated with anthocyanin formation in this variant. At the level of enzymes, significantly lower FHT and DFR activities, but not of phenylalanine ammonia-lyase (PAL) and CHS/CHI, were observed in the fruit skins of albino bilberries. A strong increase in relative hydroxycinnamic acid derivative concentrations indicates the presence of an additional bottleneck in the general phenylpropanoid pathway at a so far unknown step between PAL and CHS.

## Introduction

Bilberry (*Vaccinium myrtillus* L.) is a well-known deciduous dwarf shrub growing mostly in cool temperate regions and mountain areas of Europe and Asia. The berries are a rich source of various phenolic compounds, including large amounts of anthocyanins [1–3]. This flavonoid subclass provides the main red, violet and blue pigments in flowers and fruits, in which they act as insect and animal attractants, possess protective roles against various biotic and abiotic stresses and also provide benefits for human health [4]. In fruits, anthocyanins are predominantly found in the vacuoles of skin, although anthocyanins can also be found in the pulp in some berries [5, 6].

Differences in the composition and content of anthocyanins and other polyphenols in fruits are the consequence of complex metabolic networks, regulated by genetic, developmental and environmental factors [7–11]. Flavonoids are synthesized via the phenylpropanoid/flavonoid pathway, the main steps of which are well known [4, 12, 13]. The regulation of this pathway occurs by the interaction of various transcription factors; R2R3 MYB, basic helix–loop–helix (bHLH), WD40-like proteins and MADS-box genes [7, 14, 15]. R2R3-MYB transcription factors are the key switches for secondary metabolite gene regulation and are therefore important regulators of anthocyanin, proanthocyanidin and flavonol biosynthesis in plants [14]. They are known to regulate the expression of *chalcone synthase* (*CHS*), *flavanone 3-hydroxylase* (*FHT*), *dihydroflavonol 4-reductase* (*DFR*), *anthocyanidin synthase* (*ANS*) and other flavonoid pathway genes in various plant parts (leaves, flowers and fruits) of different horticultural plants such as anthurium [16], apple [17], bog bilberry [18] etc. One of the factors that determines whether flower and fruit color is also the competition of flavonoid 3’-hydroxylase (F3’H) and flavonoid 3’, 5’-hydroxylase (F3’5’H) with DFR for substrates, as well as substrate specificity of DFR itself [19, 20]. This results in different compositions of pelargonidin (orange), cyanidin (red) and delphinidin (blue) derivatives. Mutations of structural and regulatory genes can also result in different yellow or white anthocyanin-free phenotypes [19, 21]. Such color mutants have always been valuable study objects to obtain insights into the regulation of anthocyanins in nature [22]. Previous studies of rare berry colors of other *Vaccinium* species have thus provided a detailed insight into the gene expression of structural and regulatory genes of the anthocyanin pathway [18, 23, 24].

A downregulation of the structural genes of the anthocyanin pathway in *Vaccinium* seems to be correlated with strongly decreased expression of the transcription factors *VuMYBPA1* and *VuMYBR3* and a moderately but significantly lowered gene expression of *VuTDR4* and *VuMYBC2* [18]. *VuMYBPA1* is a R2R3 MYB transcription factor with a presumed role in anthocyanin formation in *Vaccinium* species [18], although a closely related transcription factor in *Vitis vinifera* (*VvMYBPA1*) rather controls proanthocyanidin formation in grapevine [25]. *PhMYB27* and *VvMYBC2-L1* are negative regulators of the pathway to anthocyanidins and proanthocyanidins in petunia and grapevine, respectively [26, 27]. *TDR4* has recently been suggested to play an important role in the accumulation of anthocyanins in *Vaccinium* species [24] but final evidence is not yet available.

We recently discovered a rare Slovenian wild-growing albino bilberry (*V*. *myrtillus*) differing from blue wildtypes in terms of fruit quality parameters such as fruit weight, color and primary and secondary metabolite composition [28]. Common blue ripe bilberries, typically accumulate high amounts of blue colored delphinidin- and red colored cyanidin-derived pigments and additionally contain significant levels of hydroxycinnamic acid derivatives, flavanols and flavonol glycosides. Our albino variant generally showed strongly reduced polyphenol content, which was in contrast to a recently reported albino variant of the closely related wild-type bog bilberries (*Vaccinium uliginosum* L.) which had reduced amounts of anthocyanins but unchanged flavonol and flavanol levels in comparison common blue berries and thus possesses a bottleneck in the late anthocyanin pathway [18]. Our almost anthocyanin-free albino fruits contained predominantly hydroxycinnamic acid derivatives and only moderate amounts of flavanols and flavonol glycosides.

We here present a detailed study of this albino variant of *V*. *myrtillus* and show how the two color types of wild bilberry (albino and blue) vary in their relative expressions of selected structural and regulatory genes of anthocyanin biosynthesis. In addition, we measured the specific activities of selected enzymes and concentrations of primary and secondary metabolites. We particularly focused on the polyphenol metabolism in berry skins, since previous studies have indicated that some key genes that are related to anthocyanin accumulation are expressed mainly in the skin [29, 30].

## Materials and methods

### Plant material

Wild blue and albino bilberry fruits were collected at the fully ripe stage on 11 June 2015 from native population in a forest near Žiri, 40 km west of Ljubljana, Slovenia. Blue and albino type fruits were randomly collected (approx. 300 and 150 g of fruit, respectively) and only undamaged fruits were selected for the analysis. Immediately after harvest, berry skin was separated from the pulp, shock-frozen in liquid nitrogen, and stored at -80°C until analyses of enzyme activities, relative expression and secondary metabolite concentrations. For analysis of primary metabolites, whole berries were stored at -20°C.

### Experimental design

For extraction of sugars, organic acids and phenolic compounds for each bilberry type (blue and albino), five biological replications were carried out (n = 5), while for enzyme and gene expression analysis three biological replications were carried out (n = 3). Each replication included at least 15 berries. For enzyme assays, each replication was analyzed with 2 technical replications each. RT-qPCR was carried out in biological triplicates with three technical replications each. Primary metabolites were analyzed in whole bilberry fruit, while secondary metabolites in addition to gene expression and enzyme activity were analyzed only in berry skin.

### Chemicals

For the determination of primary and secondary metabolites, same reference compounds were used as previously reported in our study [28]. Methanol, ethanol, and gallic acid were obtained from Sigma-Aldrich Chemie (Steinheim, Germany) and sodium carbonate from Merck (Darmstadt, Germany). The Folin-Ciocalteu phenol reagent and the solvents for the mobile phases, HPLC-MS grade acetonitrile and formic acid, was purchased from Fluka Chemie (Buchs, Switzerland). Water for the mobile phase was double distilled and purified with the Milli-Q system (Millipore, Bedford, MA, USA). L-(U-^14^C) Phenylalanine and (2-^14^C)-malonyl-coenzyme A were obtained from Amersham International (Freiburg, Germany). (^14^C)-Labeled flavonoids naringenin, dihydrokaempferol (DHK), dihydromyricetin (DHM), and dihydroquercetin (DHQ) were prepared as described previously [20, 31].

### Extraction and determination of sugars and organic acids

Berries (2 g) were ground to a fine paste in a mortar, homogenized with 8 mL of double distilled water and left for 30 min at room temperature. After the extraction, the homogenate was centrifuged and the supernatant was filtered and transferred into a vial. The further analysis was made as described by our previous study [28]. The results were expressed in mg g^-1^ FW.

### Extraction and determination of individual phenolic compounds

Berry skins were ground to a fine paste in a mortar chilled with liquid nitrogen, and 0.5 g were extracted with 6 (albino) or 8 mL (blue bilberries) methanol containing 3% (v/v) formic acid in a cooled ultrasonic bath for 1 h. Skin extracts were centrifuged and each supernatant was filtered and transferred to a vial prior to injection into the HPLC system. The further analysis was made as described by our previous study [28]. The results were expressed in mg kg^-1^ FW.

### Determination of total phenolic content

The extraction of skin berry samples for the determination of total polyphenols was carried out according to the same protocol as for individual polyphenols. Total polyphenol concentrations of extracts were estimated by the Folin-Ciocalteu phenol reagent method [32] and expressed as gallic acid equivalents in mg kg^−1^ FW. Absorption was measured in five replications.

### Extraction and enzyme assays

Shock-frozen bilberry skin was ground to powder with liquid nitrogen. A total of 0.20 g fine skin powder was homogenized with 0.20 g quartz sand, 0.20 g Polyclar AT, and 3 mL extraction buffer (prepared as described by Thill et al. [33]. The homogenate was centrifuged for 10 min at 4°C and 13.000 x *g*. To remove low molecular compounds, 400 μL of supernatant were passed through a gel chromatography column (Sephadex G25 medium). The protein solution eluted in the excluded volume of the column (crude extract) was used for enzyme assays.

Enzyme assays were performed as described previously [34] using the assay conditions optimized for bilberry skin (S1 Table). The assays were incubated for 15 min at 30°C. To determine the specific enzymatic activity, a modified Lowry method for protein determination [35] with BSA as a standard was used. Specific activities of PAL (phenylalanine ammonia-lyase), CHS/CHI, FHT and DFR were calculated and expressed as kat kg^-1^ protein.

### Gene expression studies

Total RNA was prepared according to Chang et al. [36] and subsequently used for the isolation of mRNA via the μMACS mRNA isolation kit (Miltenyi Biotec, Auburn, CA, USA). cDNA was prepared using the RevertAid H Minus MuLV reverse transcriptase (Fermentas Life Science, St. Leon-Rot, Germany) with the oligo(-dT) anchor Primer GACCACGCGTATCGATGTCGAC(T)_16_V.

Relative gene expressions of *ANR* (*anthocyanidin reductase*), *ANS*, *CHS*, *CHI*, *DFR*, *F3’5’H*, *FHT*, *FGT* (*flavonoid 3-O-glycosyltransferase*), *LAR* (*leucoanthocyanidin reductase*), *MYBC2*, *MYBPA1*, *MYBR3* and *TDR4* in comparison to the *glycerine aldehyde 3-phosphate dehydrogenase* (*GAPDH*) control gene were analyzed by qPCR using a StepOnePlus system and the SYBR Green PCR Master Mix (Applied Biosystems, Darmstadt, Germany) according to the supplier’s instruction. Primers for RT-qPCR were used as published elsewhere for *V*. *uliginosum* [18]. Specificity was confirmed by melting curve analysis. All primers showed an efficiency between the limits of 90 and 110%. Primer suitability of *V*. *uliginosum* for the orthologous genes of *V*. *myrtillus* was confirmed by sequencing of the amplification products. Additionally, we designed primers for *V*. *myrtillus FHT*, by using a public available sequence (NCBI AY123766). A summary of the primers used in this study are provided in S2 Table.

Differences between the cycle threshold (*Ct*) of the target gene and the *GAPDH* gene were used to obtain relative transcript levels of the target gene, and calculated as 2 exp-(Ct_target_ – Ct_*GAPDH*_). The efficiency of the RT-qPCR-reaction was determined on the basis of standard curves which were obtained by applying different DNA concentrations. Results were calculated in relation to the control gene.

### Statistical analysis

Results were evaluated with the Statgraphics Centurion XV.II program (Statpoint Technologies Inc., Warrenton, VA, USA). The significance of the type on the content of primary and secondary metabolites, relative expressions of flavonoid genes, transcription factors and enzyme activities were tested using One-Way ANOVA. Differences between forms were tested with the LSD test at a significance level of 0.05.

## Results

### Relative expression of structural and regulatory genes of the anthocyanin pathway

**Fig 1:**
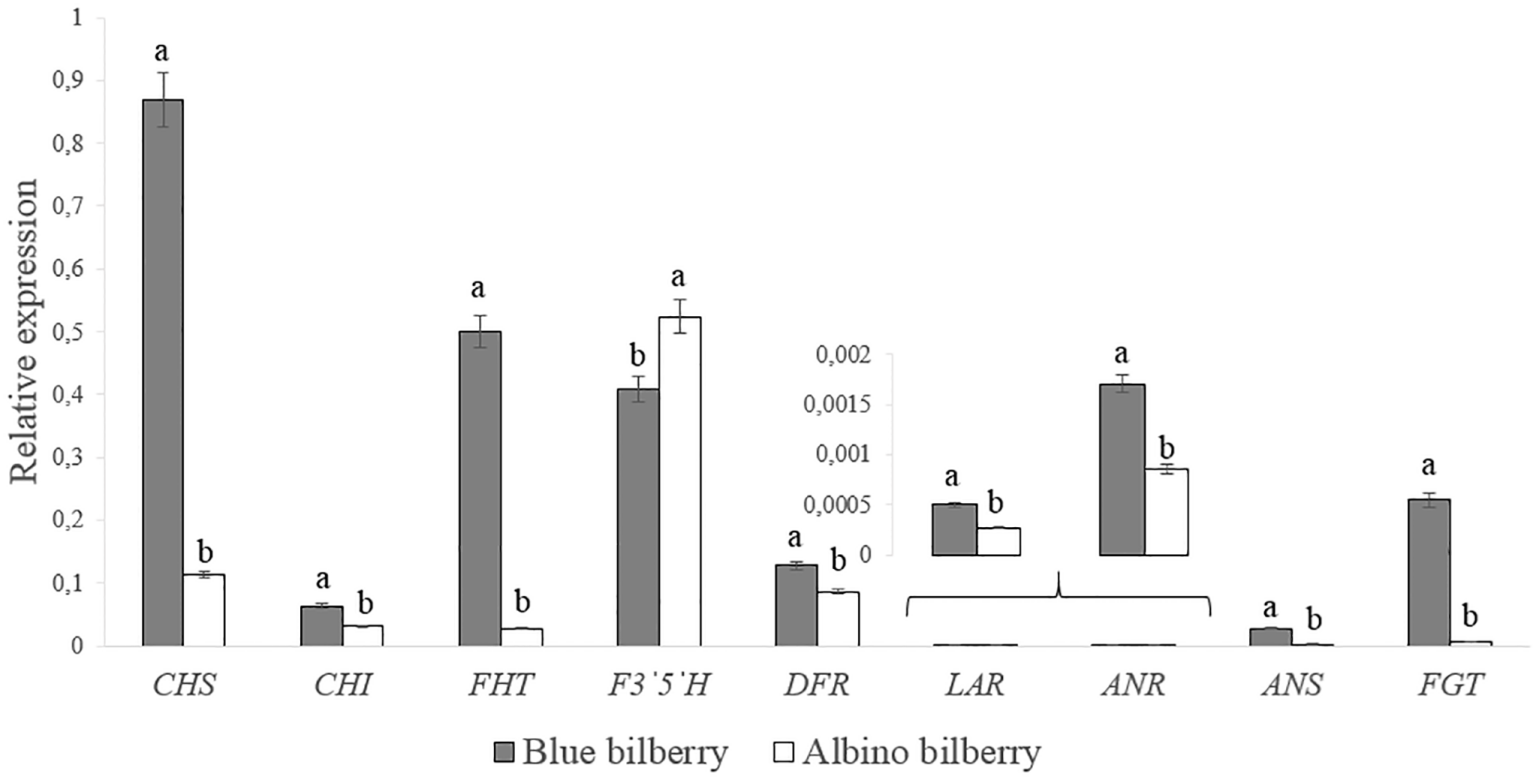
Relative expression of genes from the anthocyanin pathway (*CHS*, *CHI*, *FHT*, *F3’5’H*, *DFR*, *LAR*, *ANR*, *ANS* and *FGT*) of blue and albino bilberry normalized to *GAPDH*. Different letters (a, b) above the columns denote significant differences among bilberries (LSD test *P* < 0.05).

**Fig 2:**
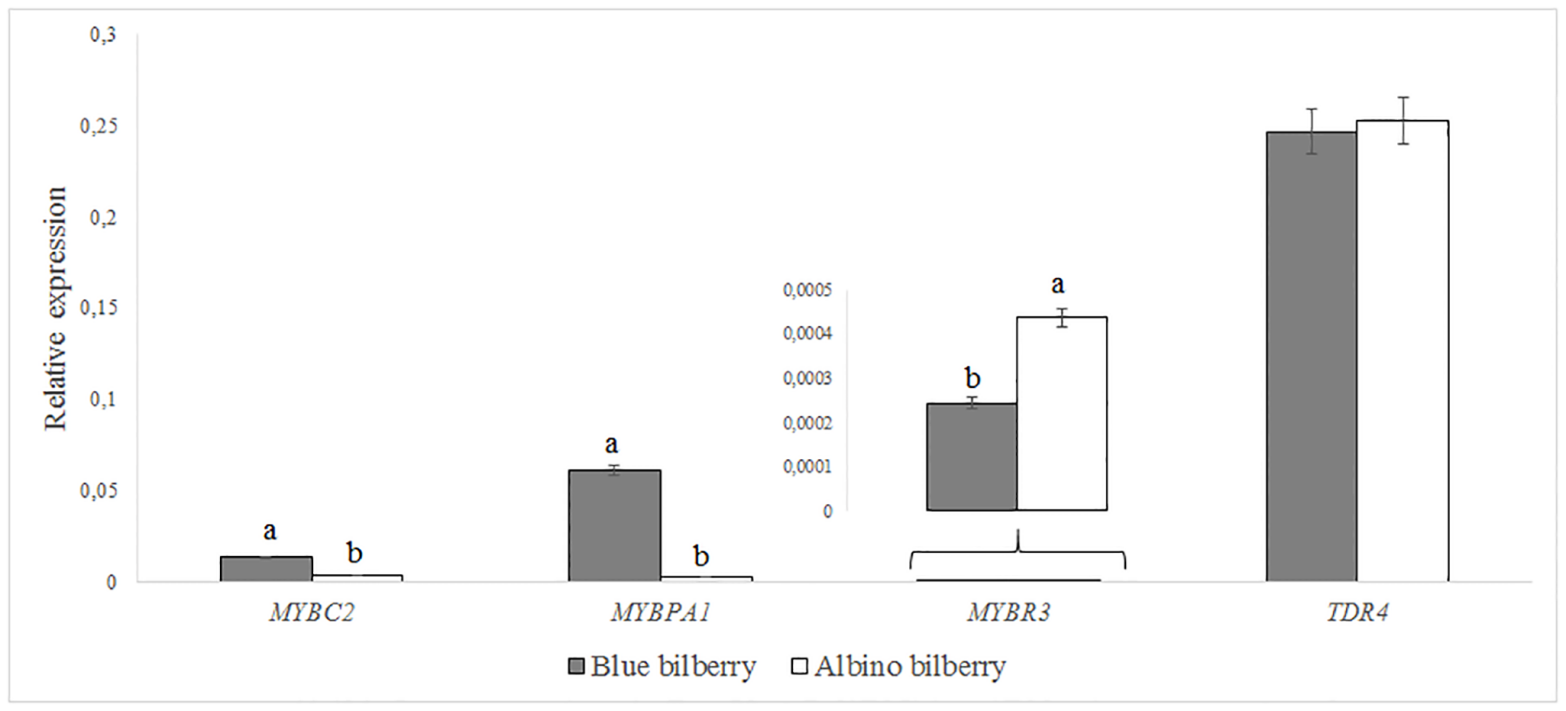
Relative expression of transcription factors (*MYBC2*, *MYBPA1*, *MYBR3* and *TDR4*) of blue and albino bilberry normalized to *GAPDH*. Different letters (a, b) above the columns denote significant differences among bilberries (LSD test *P* < 0.05).

The relative expression of the structural genes *CHS*, *CHI*, *FHT*, *F3’5’H*, *DFR*, *LAR*, *ANR*, *ANS* and *FGT* of the flavonoid pathway and of four transcription factors previously described as influencing anthocyanin accumulation in *Vaccinium* was determined in the blue and albino bilberry skins (Figs 1 and 2). The latter included transcription factors of the R2R3 and R3 MYB family (*MYBC2*, *MYBPA1* and *MYBR3*) and the SQUAMOSA class transcription factor *TDR4* (Fig 2). After evaluation of nine candidates of housekeeping genes with respect to expression stability and quality of signals obtained with published primer sequences for *Vaccinium* sp. [37], *GAPDH*, *tubulin ß* and *clathrin adaptor complexes subunit family protein* remained as suitable housekeeping genes. Considering the fact that only fruits from the same location and grown under identical conditions were analyzed, we used *GAPDH* as single housekeeping gene for the studies.

Most of the primers were derived from studies on *V*. *uliginosum* but could be used for our studies as well. The successful application of RT-qPCR primers of *V*. *uliginosum* for genes of the closely related *V*. *myrtillus* showed that the interspecific usage of primers can be successfully applied if sequence information is lacking.

In comparison to common blue berry skins of *V*. *myrtillus*, the albino type showed strongly decreased gene expression of all tested structural genes, with the exception of *F3’5’H* expression, which was only 1.3-fold higher in albino bilberry and thus almost unchanged (Fig 1). The most affected genes were *FGT* (33-fold lower), *FHT* (18-fold lower gene expression), *ANS* (11-fold lower) and *CHS* (7.6-fold lower), while the other structural genes (*CHI*, *DFR*, *LAR* and *ANR*) showed only a 1.5–2.1-fold lower gene expression in albino bilberry (Fig 1).

Among transcription factors, the highest difference between *V*. *myrtillus* color types, with almost 22-fold lower relative expression level for albino bilberry, was measured for *MYBPA1* (Fig 2). In addition, albino bilberry had 3.7-fold lower relative expression of *MYBC2* but a 1.8-fold higher relative expression of *MYBR3* (Fig 2). For *TDR4* gene expression, no significant differences were observed between blue and albino *V*. *myrtillus* berries (Fig 2).

### Specific activities of selected flavonoid enzymes

**Fig 3:**
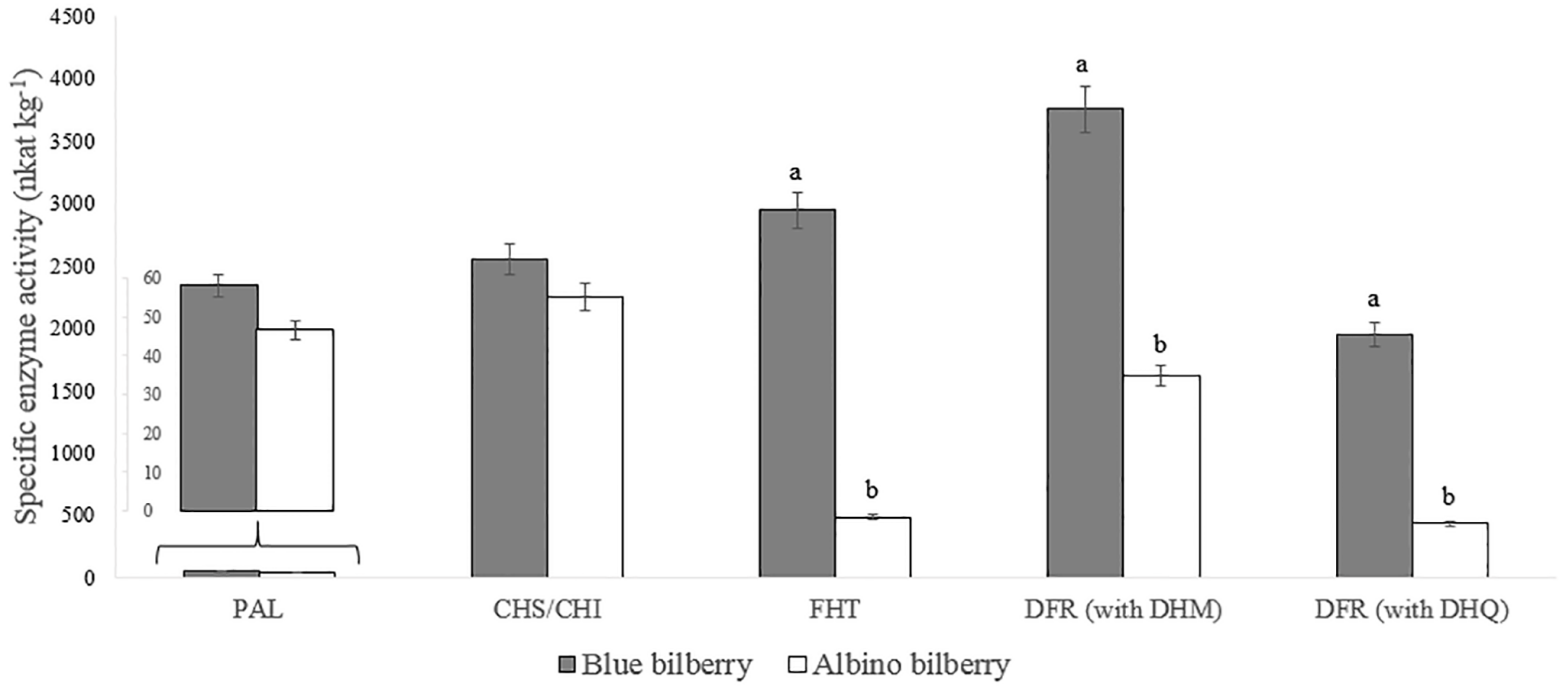
Specific enzyme activities (PAL, CHS/CHI, FHT and DFR (with DHQ and DHM as substrates) (nkat kg^-1^ protein) of blue and albino bilberry. Different letters (a, b) above the columns denote significant differences among bilberries (LSD test *P* < 0.05).

In addition to the gene expression studies, we also measured for the first time the activities of the enzymes of the main pathway to anthocyanins, PAL, CHS/CHI, FHT and DFR (Fig 3). ANS could unfortunately not be included, since its activity can so far only be measured with recombinant enzymes [13].

Significant differences between the two *V*. *myrtillus* color types were observed for FHT and DFR activities, but not for PAL and CHS/CHI (Fig 3). A 6.1-fold lower specific activity of FHT was measured in the albino type (Fig 3). DFR enzyme activities were tested with DHK, DHQ and DHM as substrates (S1 Table). Enzyme preparations from *V*. *myrtillus* did not convert DHK; moreover DFR preferred DHM over DHQ (1.9-fold and 3.7-fold higher conversation rates for blue and albino bilberry, respectively). Higher differences in DFR enzyme activities among the tested bilberries were measured with DHQ, with a 4.5-fold lower activity in the albino bilberry (Fig 3). DFR with DHM as a substrate had only a 2.3-fold lower activity in the albino compared to the blue bilberry (Fig 3).

### Phenolic profile of bilberry fruit skins

**Fig 4:**
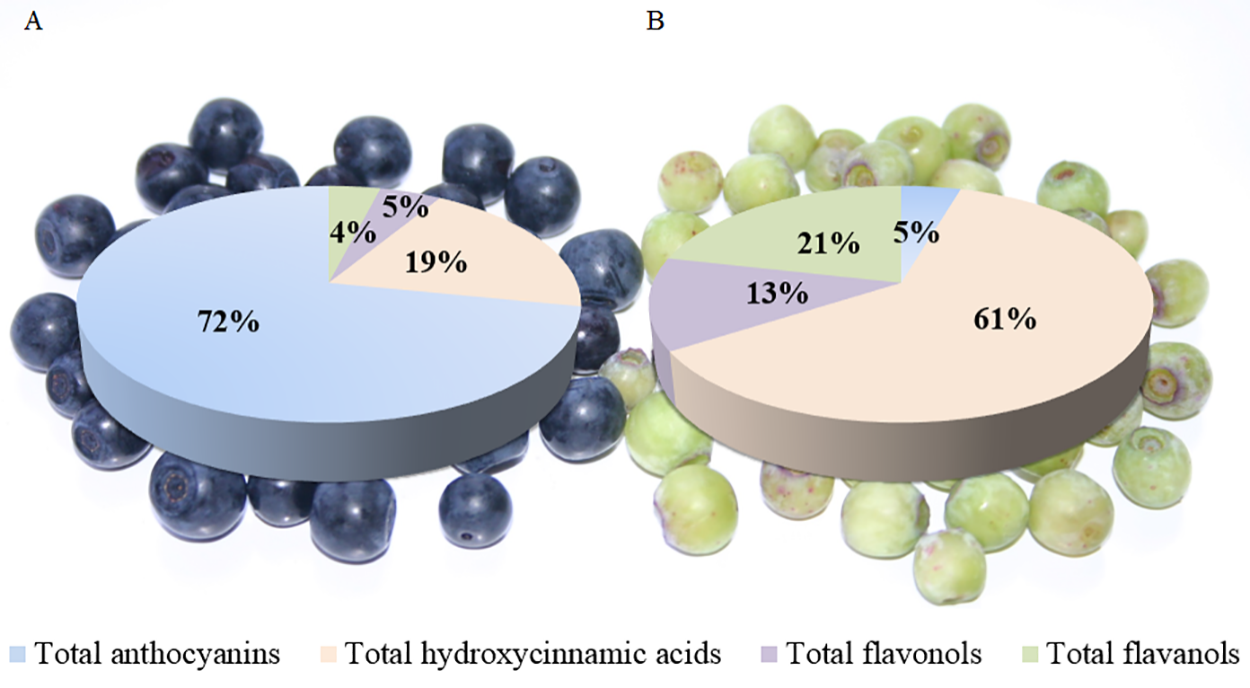
Relative levels of the main polyphenol classes of bilberry fruit skins. (A) Blue bilberry. (B) Albino bilberry.

To provide a more detailed insight into the complex flavonoid pathway in the two *V*. *myrtillus* types, the composition and content of primary and secondary metabolites were additionally analyzed (S3–S5 Tables). Since detailed phenolic characterization of both bilberry fruits was reported in our previously published work [28], in this study we analyzed only fruit skin phenolics, which are presented in S4 and S5 Tables. Fig 4 provides an overview on the relative levels of the main polyphenol classes of both bilberry skins.

In the albino type, the majority of polyphenols were found to be hydroxycinnamic acid derivatives (61%), followed by flavanols (21%) and flavonol glycosides (13%), whereas total anthocyanins contributed only 4.5% (Fig 4 and S4 Table). The polyphenol spectrum of blue *V*. *myrtillus* berry skins, in contrast, consisted of 72% anthocyanins, 19% hydroxycinnamic acid derivatives and 4–5% of flavanols and flavonol glycosides (Fig 4 and S4 Table). Due to the very low polyphenol concentrations (7-fold lower total phenolic content in albino compared to blue bilberry), the absolute amounts of each polyphenol class were higher in blue than albino type berry skins (S5 Table). Although there were differences in the individual composition of polyphenol classes (S4 Table), no striking qualitative differences could be found between the two color types. Even in the anthocyanin spectrum, albino *V*. *myrtillus* berry skins accumulated the same 15 anthocyanins, albeit in drastically lower amounts (151-fold lower) (S5 Table).

**Fig 5:**
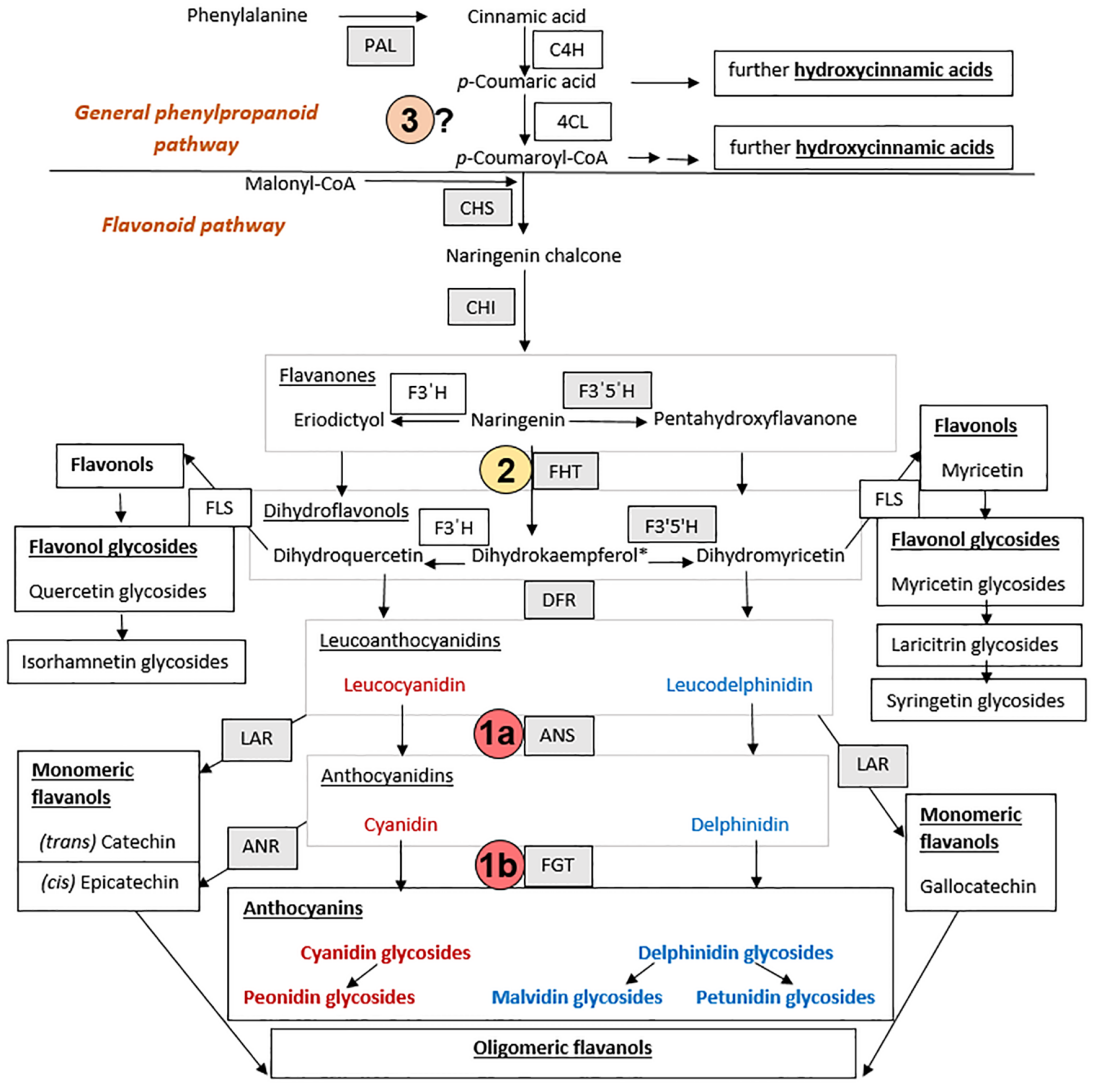
Simplified flavonoid biosynthesis pathway (combined studies of gene expression, enzyme activity and metabolite analysis) leading to anthocyanin accumulation in *Vaccinium myrtillus* L. Abbreviations: ANR, anthocyanidin reductase; ANS, anthocyanidin synthase; C4H, cinnamate 4-hydroxylase; CHI, chalcone isomerase; CHS, chalcone synthase; 4CL, hydroxycinnamate: CoA ligase; DFR, dihydroflavonol reductase; F3′H, flavonoid 3′-hydroxylase; F3′5′H, flavonoid 3′, 5′-hydroxylase; FGT, flavonoid-3-*O*-glucosyltransferase; FHT, flavanone 3-hydroxylase; FLS, flavonol synthase; LAR, leucoanthocyanidin reductase; PAL, phenylalanine ammonia lyase. *leading to small amounts of kaempferol-3-*O*-glucuronide. The three bottlenecks identified in the anthocyanin pathway of the albino type berry skins are numbered with 1 (a, b: ANS, FGT), 2 (FHT) and 3 (a so far unknown step located in the general phenylpropanoid pathway between PAL and CHS). Grey shaded boxes, enzymes/genes analyzed in our work.

An overview of the flavonoid pathway in *V*. *myrtillus* according to our results obtained in this study is summarized in Fig 5.

## Discussion

In this study we characterized for the first time the flavonoid pathway of an albino type of *V*. *myrtillus* found in Slovenia [28] by measuring the expression of a range of structural and regulatory genes and selected enzyme activities correlated with the polyphenols accumulated in the berry skins. Whereas the flavonoid pathway is well established and structural genes can be found in the databases, if not always for *V*. *myrtillus* then at least for another *Vaccinium* species, the knowledge on the regulatory genes influencing the formation of polyphenols, especially flavonoids is fragmented. We included, however, a number of transcription factors, that have been previously assumed to play a role in the anthocyanin pathway in any of the *Vaccinium* species, to shed first light on the question if our *V*. *myrtillus* albino variant can provide novel insights into the pathway or if it is similar to other *Vaccinium* color types previously reported.

Our study revealed significant differences in the expression levels of key structural genes and specific enzyme activities of the anthocyanin pathway between albino and blue colored *V*. *myrtillus* fruit skins. Key structural genes showed considerably lower expression levels in albino bilberry skins, compared to common blue type. Among them, the most affected genes were *FGT*, *FHT*, *ANS* and *CHS*, followed by *CHI*, *DFR*, *LAR* and *ANR*, while *F3’5’H* gene expression was slightly higher in albino *V*. *myrtillus* (Fig 1). This is in line with reports on albino *V*. *uliginosum* and *V*. *myrtillus*, in which the whole pathway with the exception of *F3’5’H* was strongly downregulated in ripe fruits [18, 23]. It also confirms that *F3’5’H* is regulated separately as suggested from earlier studies of *V*. *uliginosum* [18].

Our albino *V*. *myrtillus*, however, showed interesting differences in the expression of transcription factors compared to other albino fruits of *V*. *uliginosum* and *V*. *myrtillus*. We observed significantly lower relative expression levels of *MYBPA1* and *MYBC2*, but higher relative expression of *MYBR3* in albino compared to blue bilberry, while for *TDR4* gene expression, no differences were observed (Fig 2). A high correlation of *MYBPA1* expression with the anthocyanin pathway gene expression has largely been reported earlier [15, 16, 18, 24, 38]. Other albino *Vaccinium* fruits, however, showed a strong correlation between the expression of *TDR4* and various MYB factors, suggesting that *TDR4* plays an important role in the control of anthocyanin biosynthesis in *Vaccinium* berries [18, 24]. This was supported by virus-induced gene silencing of *TDR4*, leading to a strong reduction of anthocyanin concentrations in the berries. It seems, however, that in this experiment the flesh was much more affected than skin, since the ripe fruits had a clearly faded flesh color, despite an intense skin coloration [24]. In agreement with this, our data also suggest that *TDR4* is not necessarily correlated with anthocyanin based coloration in *V*. *myrtillus*, at least not in the skins. The moderate increase of *MYBR3* gene expression in the albino *V*. *myrtillus* type is in contrast to the observed downregulation in albino *V*. *uliginosum* [18] and to *PhMYBx* (R3-MYB) from *Petunia*, which was upregulated when plants begin to accumulate anthocyanins [39]. Although anthocyanin biosynthesis in plants usually involve R2R3 and R3 MYB activators, there are also multiple types of repressors, which are less understood [26, 39]. In *Petunia hybrida*, a putative R2R3-MYB repressor of anthocyanin synthesis *PhMYB27* was identified and in strawberry structurally similar *FaMYB1*, though there are also some other known ones [39, 40].

The albino type *V*. *myrtillus* berry skins showed comparable results as the previously reported whole bilberry fruits [28]. As expected, a pronounced shift in the composition of polyphenol classes was observed, which was also characterized by a drastically lower accumulation of total polyphenols, which was as low as 15% of the concentrations found in the blue berries (S5 Table). The low content of total polyphenols indicates the presence of a bottleneck early in the pathway, located between PAL and CHS, which was not encompassed by the enzyme and gene expression studies as the sequences from *Vaccinium* are not yet available (Fig 3). The discrepancies between CHS/CHI enzyme activity and *CHS* gene expression is most probably the result of the presence of isoenzymes as frequently observed for flavonoid enzymes [41]. In *Dahlia* x *variabilis* two phylogenetically different chalcone synthases were described sharing only 69% nucleotide sequence identity, of which one is generally present, whereas the second is specifically upregulated together with DFR and ANS during anthocyanin formation [42]. Indeed the presence of several isoforms of the genes from the anthocyanin pathway was reported for *Vaccinium* species [23]. This underpins the importance of measuring enzyme activities in addition to gene expression to obtain a better picture of the pathway.

The shift in the other polyphenol classes correlated nicely with the observed enzyme activities and gene expression levels. In general, a lower FHT and DFR activity in albino bilberry was found and correlated with the higher relative *FHT* and *DFR* expression in blue bilberry (Figs 1 and 2). Additionally, the bottleneck created by low *ANS* and *FGT* expression resulted in drastically lower anthocyanin accumulation in albino skins (S5 Table), as described for other plants [18, 19, 43]. In a previous study [23], a reduction in levels of *DFR* and *ANS* in pink and white colored bilberries was also demonstrated but their transcript abundance was not measured by RT-qPCR. The increase in the relative epicatechin concentrations in our albino *V*. *myrtillus* berry skins (S4 Table) may reflect the redirection of anthocyanidin formation to epicatechin formation, since *ANR* and *ANS* expression were reduced to a minor extent compared to *FGT*. The slightly increased *F3’5’H* expression in albino *V*. *myrtillus* berry skins, however, was not reflected in elevated amounts of 3’, 4’, 5-hydroxylated anthocyanins (delphinidin type) or flavonols (myricetin type). In our albino type *V*. *myrtillus*, FHT and DFR seem to form a two-step bottleneck, with a lower flux at the first FHT step. The low amounts of dihydroflavonols formed are common substrates for DFR and flavonol synthase (FLS). Substrate competition between DFR (anthocyanin pathway) and FLS (side branch to flavonols) has been reported previously for many plants [19, 33, 44]. In detail, enzyme preparations from *V*. *myrtillus* did not convert DHK, which is in line with the absence of pelargonidin derived anthocyanins. A changed flavonoid flux due to the DHM preference of DFR may provide an explanation of the relatively lower myricetin concentrations in the albino type compared to the blue (S4 Table). Although DFR preferred DHM over DHQ, higher differences in DFR enzyme activities among the tested bilberries were measured with DHQ (Fig 2). The 4.5-fold higher activity in blue bilberry with DHQ compared with a 2.3-fold higher activity with DHM confirms the presence of DFR isoenzymes in bilberry that show different substrate specificities, as recently shown for *Fragaria* species [20]. If only one isoform were to be present, a similar reduction in the conversion of DHM and DHQ would be expected. Actually at least two different types of DFRs (Accession numbers AY780883, AF483836) have been identified in the closely related *V*. *macrocarpon* [45, 46].

## Conclusions

This study provides an in-depth characterization of gene expression and enzyme activities and metabolites of the polyphenol pathway of an albino type of wild *V*. *myrtillus* recently found in Slovenia. We identified three bottlenecks in the anthocyanin pathway in the berry skins of the albino type at the level of (1) ANS/FGT, (2) FHT and (3) a so far unknown step located in the general phenylpropanoid pathway between PAL and CHS. The latter is clearly reflected by drastically lower total polyphenol concentrations in the albino *V*. *myrtillus* berry skins and a shift in the polyphenol profile towards a prevalent presence of hydroxycinnamic acids and increased relative (but not absolute) concentrations of flavanols and flavonols. Thus our albino type adds another model type for studying the anthocyanin pathway and its regulation in *Vaccinium* fruits, which particularly offers the possibility to focus on the general phenylpropanoid pathway upstream of flavonoid formation. Further work will focus on the identification of the bottleneck located in the general phenylpropanoid pathway, the identification of isoenzymes of the flavonoid pathway and putative differences in their substrate specificity as well as transcriptome studies to identify so far unknown further regulatory genes involved in the formation of anthocyanins in *Vaccinium* species.

## Supporting information

S1 Table Optimized conditions for assays of main enzymes in the flavonoid pathway of bilberry. (PDF)

S2 Table List of primers used in the qPCR studies. (PDF)

S3 Table Content levels of individual and total sugars, organic acids (mg g^-1^ FW) and sugar/organic acid ratio of blue and albino bilberry fruit. (PDF)

S4 Table Relative levels of individual phenolic compounds within flavonoid class and relative level of flavonoid class regarding total analyzed phenolics of blue and albino bilberry skins. (PDF)

S5 Table Content levels of total anthocyanins, flavanols, flavonols, hydroxycinnamic acid derivatives, hydroxybenzoic acid derivative and total phenolic content (mg kg^-1^ FW) of blue and albino bilberry skins. (PDF)
