# Supplementary material for: A wild ‘albino’ bilberry (Vaccinium myrtillus L.) from Slovenia shows three bottlenecks in the anthocyanin pathway and significant differences in the expression of several regulatory genes compared to the common blue berry type
Source: PLoS One. 2017 Dec 22;12(12):e0190246. doi: 10.1371/journal.pone.0190246 (PMC5741254; doi:10.1371/journal.pone.0190246)
Supplement: S1 Table — (PDF) [file pone.0190246.s001.pdf]

S1 Table. Optimized conditions for assays of main enzymes in the flavonoid pathway of bilberry.

Enzyme	Crude extract (µl)	Buffer	(µl)	Substrate	(µl)	Cofactor	(µl)	Final volume (µl)	Stop reaction (µl)
PAL	40	0.1 M KPi + 0.4% Na-ascorbate, pH 8.5	55	(^{14}C)-phenylalanine (0.063 nmol)	5	-		100	200 EE*<br>10 HAC**
CHS/ CHI	40	0.1 M KPi + 0.4% Na-ascorbate, pH 7.5	50	(^{14}C)-malonyl-CoA (1.5 nmol)<br>*p*-coumaroyl-CoA (1 nmol)	5<br>5	-		100	200 EE<br>10 HAC
FHT	30	0.1 M Tris/HCl + 0.4 % Na-ascorbate, pH 7.5	60	(^{14}C)-naringenin (0.036 nmol)	-	2-oxoglutarate (1.46 mg/ml H_2O)<br>$FeSO_4 \times 7 H_2O$ (0.56 mg/ml H_2O)	5<br>5	100	70 EE<br>10 HAC
DFR	20	0.1 M KPi + 0.4% Na-ascorbate, pH 6.5	25	(^{14}C)-DHM/ DHQ/ DHK (0.036 nmol)	-	NADPH (4.18 mg/100 µl H_2O)	5	50	70 EE

*EE ethyl acetate
**HAC acetic acid
