# Supplementary material for: A wild ‘albino’ bilberry (Vaccinium myrtillus L.) from Slovenia shows three bottlenecks in the anthocyanin pathway and significant differences in the expression of several regulatory genes compared to the common blue berry type
Source: PLoS One. 2017 Dec 22;12(12):e0190246. doi: 10.1371/journal.pone.0190246 (PMC5741254; doi:10.1371/journal.pone.0190246)
Supplement: S2 Table — (PDF) [file pone.0190246.s002.pdf]

S2 Table. List of primers used in the qPCR studies.

Gene	Primer sequence	Source
VmCHS	GCTCCCTTGTTGTTCTCTGC (forward)	*V. uliginosum*[1]
	ACCCACTTGGTCTTTTGCAC (reverse)	
VmCHI	GTCTTTCCTCCGTCGATCAAAC (forward)	*V. uliginosum*[1]
	CGATCTTGCCCTTCCACTTAAC (reverse)	
VmDFR	GAAGTGATCAAGCCGACGAT (forward)	*V. uliginosum*[1]
	ATCCAAGTCGCTCCAGTTGT (reverse)	
VmANS	CTTCTACGAGGGCAAATGGA (forward)	*V. uliginosum*[1]
	GCCCACGAAATCCTAACCTT (reverse)	
VmFHT	CTGAGGACAAGAAGGACATGATTA (forward)	*V. myrtillus* AY123766
	CCGATAGGACCTTGGTTGTTAG (reverse)	
VmF3′5′H	AAGCGTATGCATAGCAAGTGG (forward)	*V. uliginosum*[1]
	TGATTCGGTGCCTTGAGAAT (reverse)	
VmLAR	CGTTGATCACAGCTTCTGTTGC (forward)	*V. uliginosum*[1]
	CGCCTTTGTCTTGGAGAGTCTT (reverse)	
VmANR	GCTGGTGTTTCTCCCACAAT (forward)	*V. uliginosum*[1]
	AAATATATGGGCGCGACAAA (reverse)	
VmUFGT	CATCCAAACCCTGTTCCCATCC (forward)	*V. uliginosum*[1]
	TCATCCCTGCCTTCAAGCTCTC (reverse)	
VmMYBC2	GCAGGCTGCAGGATTACTTC (forward)	*V. uliginosum*[1]
	TGGATCGATACCCATGTTCA (reverse)	
VmMYBPA1	ATTCAACTTCATGGCGAAGG (forward)	*V. uliginosum*[1]
	GGCATGCATTCTTATAATGAGGT (reverse)	
VmMYBR3	GAGCGAATTCAGGCATCTGT (forward)	*V. uliginosum*[1]
	CCCAAAAACCTTGAACACGA (reverse)	
VmTDR4	CACCTTGACCCTGAGAGAGC (forward)	*V. uliginosum*[1]
	GTCCACCTTGGTTTTGTTGC (reverse)	
VmGAPDH	CAAACTGTCTTGCCCCACTT (forward)	*V. uliginosum*[1]
	CAGGCAACACCTTACCAACA (reverse)	

[1]Primetta et al. 2015
