# Supplementary material for: A wild ‘albino’ bilberry (Vaccinium myrtillus L.) from Slovenia shows three bottlenecks in the anthocyanin pathway and significant differences in the expression of several regulatory genes compared to the common blue berry type
Source: PLoS One. 2017 Dec 22;12(12):e0190246. doi: 10.1371/journal.pone.0190246 (PMC5741254; doi:10.1371/journal.pone.0190246)
Supplement: S3 Table — (PDF) [file pone.0190246.s003.pdf]

S3 Table. Content levels of individual and total sugars, organic acids (mg g^{-1} FW) and sugar/organic acid ratio of blue and albino bilberry fruit.

	**Blue bilberry**	**Albino bilberry**
**Fructose**	36.48 ± 1.24 ns	37.84 ± 0.60 ns
**Glucose**	34.10 ± 1.04 ns	34.74 ± 0.47 ns
**Total sugars**	**70.58 ± 2.19 ns**	**72.58 ± 1.01 ns**
**Citric acid**	9.22 ± 0.38 a	8.00 ± 0.19 b
**Quinic acid**	8.03 ± 0.36 a	5.67 ± 0.16 b
**Malic acid**	3.15 ± 0.21 ns	2.71 ± 0.04 ns
**Shikimic acid**	0.14 ± 0.02 b	0.39 ± 0.00 a
**Total organic acids**	**20.54 ± 0.97 a**	**16.77 ± 0.39 b**
**Sugar/organic acid ratio**	**3.44 ± 0.18 b**	**4.33 ± 0.12 a**

Mean and standard errors are presented. Different letters (a–b) in rows denote statistically significant differences between the forms at $P < 0.05$ (LSD test).
