# Supplementary material for: A wild ‘albino’ bilberry (Vaccinium myrtillus L.) from Slovenia shows three bottlenecks in the anthocyanin pathway and significant differences in the expression of several regulatory genes compared to the common blue berry type
Source: PLoS One. 2017 Dec 22;12(12):e0190246. doi: 10.1371/journal.pone.0190246 (PMC5741254; doi:10.1371/journal.pone.0190246)
Supplement: S4 Table — (PDF) [file pone.0190246.s004.pdf]

S4 Table. Relative levels of individual phenolic compounds within flavonoid class and relative level of flavonoid class regarding total analyzed phenolics of blue and albino bilberry skins.

Blue bilberry	%	Albino bilberry	%
**Total anthocyanins**	**71.8**	**Total anthocyanins**	**4.5**
Delphinidin-3-*O*-galactoside	15.1	Delphinidin-3-*O*-galactoside	19.3
Delphinidin-3-*O*-arabinoside	10.7	Delphinidin-3-*O*-arabinoside	13.4
Delphinidin-3-*O*-glucoside	8.0	Delphinidin-3-*O*-glucoside	2.4
Cyanidin-3-*O*-glucoside	12.9	Cyanidin-3-*O*-arabinoside	15.4
Cyanidin-3-*O*-arabinoside	11.1	Cyanidin-3-*O*-galactoside	10.8
Cyanidin-3-*O*-galactoside	8.6	Cyanidin-3-*O*-glucoside	6.2
Petunidin-3-*O*-glucoside	9.6	Petunidin-3-*O*-glucoside	13.3
Petunidin-3-*O*-galactoside	6.6	Petunidin-3-*O*-arabinoside	7.6
Petunidin-3-*O*-arabinoside	2.4	Petunidin-3-*O*-galactoside	3.2
Malvidin-3-*O*-glucoside	5.1	Malvidin-3-*O*-glucoside	2.2
Malvidin-3-*O*-galactoside	3.8	Malvidin-3-*O*-arabinoside	2.1
Malvidin-3-*O*-arabinoside	1.2	Malvidin-3-*O*-galactoside	0.9
Peonidin-3-*O*-glucoside	4.0	Peonidin-3-*O*-galactoside	1.9
Peonidin-3-*O*-galactoside	0.6	Peonidin-3-*O*-glucoside	1.0
Peonidin-3-*O*-arabinoside	0.3	Peonidin-3-*O*-arabinoside	0.2
**Total flavanols**	**4.0**	**Total flavanols**	**21.4**
Epicatechin	35.3	Epicatechin	58.8
Catechin	20.6	Catechin	2.7
Procyanidin trimer	22.3		
Procyanidin dimer	19.7	Procyanidin dimer	33.6
Gallocatechin	2.1	Gallocatechin	5.0
**Total flavonols**	**4.7**	**Total flavonols**	**12.6**
Quercetin-3-*O*-glucuronide	28.6	Quercetin-3-*O*-glucuronide	61.7
Quercetin-3-*O*-galactoside	25.9	Quercetin-3-*O*-galactoside	27.0
Quercetin-3-*O*-glucoside	9.3	Quercetin-3-*O*-glucoside	3.0
Quercetin-3-*O*-rhamnoside	1.9	Quercetin-3-*O*-rhamnoside	1.1
Myricetin hexoside 2	6.4	Kaempferol-3-*O*-glucuronide	2.3
Myricetin hexoside 1	4.4	Myricetin pentoside 1	0.9
Myricetin	3.8		
Myricetin pentoside 1	0.7	Myricetin hexoside 2	0.5
Myricetin-3-*O*-glucuronide	0.7	Myricetin-3-*O*-rhamnoside	0.4
Myricetin-3-*O*-rhamnoside	0.7	Myricetin-3-*O*-glucuronide	0.2
Myricetin pentoside 2	0.1	Myricetin hexoside 1	0.1
Laricitrin-3-*O*-glucoside	10.0	Myricetin pentoside 2	0.1
Laricitrin-3-*O*-galactoside	0.04		
Laricitrin-3-*O*-glucuronide	0.01	Syringetin-3-*O*-galactoside	0.8
Syringetin-3-*O*-galactoside	4.9	Syringetin-3-*O*-glucoside	0.4
Syringetin-3-*O*-glucoside	0.2	Laricitrin-3-*O*-glucuronide	0.6
Kaempferol-3-*O*-glucuronide	1.9	Laricitrin-3-*O*-glucoside	0.3
Isorhamnetin-3-*O*-glucoside	0.4	Isorhamnetin-3-*O*-glucoside	0.4
Isorhamnetin-3-*O*-galactoside	0.02	Isorhamnetin-3-*O*-galactoside	0.3
**Total hydroxycinnamic acid derivatives**	**19.4**	**Total hydroxycinnamic acid derivatives**	**61.2**
trans-5-Caffeoylquinic acid	86.3	*trans*-5-Caffeoylquinic acid	82.9
Caffeic acid derivative 1	9.8	Caffeic acid derivative 1	3.5
		Caffeic acid derivative 2	0.7
Caffeic acid	0.2		
cis-5-Caffeoylquinic acid	0.1	*cis*-5-Caffeoylquinic acid	0.3
Caffeic acid derivative 3	0.04	Caffeic acid derivative 3	0.2
Coumaroyl iridoid isomer 3	1.3	Coumaroyl iridoid isomer 3	4.8
Coumaroyl iridoid isomer 2	0.7	*p*-Coumaric acid hexoside	3.0
Coumaroyl iridoid isomer 1	0.3	5-*p*-Coumaroylquinic acid 1	2.6
5-*p*-Coumaroylquinic acid 2	0.3	Coumaroyl iridoid isomer 2	0.8
p-Coumaric acid hexoside	0.1	5-*p*-Coumaroylquinic acid 2	0.4
Coumaric acid derivative 1	0.1	Coumaric acid derivative 1	0.3
Coumaric acid derivative 3	0.1	Coumaroyl iridoid isomer 1	0.2

5-*p*-Coumaroylquinic acid 1<br>Coumaric acid derivative 2	0.04<br>0.03	Coumaric acid derivative 3	0.2
5-Feruloylquinic acid	0.4		
**Hydroxybenzoic acid derivative**	**0.03**	**Hydroxybenzoic acid derivative**	**0.3**
Depside	100%	Depside	100%
