# Supplementary material for: A wild ‘albino’ bilberry (Vaccinium myrtillus L.) from Slovenia shows three bottlenecks in the anthocyanin pathway and significant differences in the expression of several regulatory genes compared to the common blue berry type
Source: PLoS One. 2017 Dec 22;12(12):e0190246. doi: 10.1371/journal.pone.0190246 (PMC5741254; doi:10.1371/journal.pone.0190246)
Supplement: S5 Table — (PDF) [file pone.0190246.s005.pdf]

S5 Table. Content levels of total anthocyanins, flavanols, flavonols, hydroxycinnamic acid derivatives, hydroxybenzoic acid derivative and total phenolic content (mg kg^{-1} FW) of blue and albino bilberry skins.

	**Blue bilberry**	**Albino bilberry**
Delphinidin-3-*O*-galactoside	1092.4 ± 100.7 a	9.23 ± 2.33 b
Cyanidin-3-*O*-glucoside	931.6 ± 6.12 a	2.98 ± 0.69 b
Cyanidin-3-*O*-arabinoside	800.3 ± 58.5 a	7.38 ± 1.67 b
Delphinidin-3-*O*-arabinoside	770.8 ± 41.1 a	6.42 ± 14.7 b
Petunidin-3-*O*-glucoside	694.6 ± 50.6 a	6.38 ± 1.44 b
Cyanidin-3-*O*-galactoside	617.0 ± 32.9 a	5.15 ± 1.18 b
Delphinidin-3-*O*-glucoside	576.0 ± 49.7 a	1.16 ± 0.34 b
Petunidin-3-*O*-galactoside	477.6 ± 44.4 a	1.52 ± 0.36 b
Malvidin-3-*O*-glucoside	366.2 ± 47.7 a	1.06 ± 0.39 b
Peonidin-3-*O*-glucoside	288.0 ± 51.1 a	0.46 ± 0.15 b
Malvidin-3-*O*-galactoside	274.8 ± 48.7 a	0.46 ± 0.15 b
Petunidin-3-*O*-arabinoside	170.8 ± 20.7 a	3.64 ± 1.59 b
Malvidin-3-*O*-arabinoside	86.6 ± 10.5 a	1.02 ± 0.29 b
Peonidin-3-*O*-galactoside	42.6 ± 5.16 a	0.90 ± 0.39 b
Peonidin-3-*O*-arabinoside	25.4 ± 3.39 a	0.08 ± 0.04 b
**Total anthocyanins**	**7218 ± 183 a**	**47.85 ± 11.3 b**
**Total flavanols**	**397.4 ± 61.2 a**	**225.5 ± 36.8 b**
**Total flavonols**	**473.1 ± 34.5 a**	**133.3 ± 38.4 b**
**Total hydroxycinnamic acid derivatives**	**1961 ± 82.0 a**	**645.4 ± 128 b**
**Hydroxybenzoic acid derivative**	**3.48 ± 0.29 ns**	**2.74 ± 0.26 ns**
**Total phenolic content**	**10995 ± 376 a**	**1603 ± 189 b**

Mean and standard errors are presented. Different letters (a–b) in rows denote statistically significant differences between the forms at $P < 0.05$ (LSD test).
